# Book and Pamphlet Notices

**Published:** 1859-01

**Authors:** 


### BOOK AND PAMPHLET NOTICES.

The Transactions of the American Medical Association. Instituted 1847. Vol. II. 1858.

This volume of the Transactions is rather larger than the last. It contains over one thousand pages, and is bound in the same permanent manner as its predecessors of the last three or four years.

After the minutes come the Report of the Committee on Publication and Treasurer, which are followed by the different scientific and literary papers of the President and contributors of the Association.

The first of these is the address of Prof. P. F. Eve, of Nashville, the retiring President. An excellent address, alike honorable to the profession and its author. Next follow: Report on the Medical Topography and the Epidemic Diseases of Kentucky, by W. L. Sutton, M. D., Georgetown, Ky.; Report on the Topography and Epidemic Diseases of New Jersey and the treatment thereof, by Lyndon A. Smith, M.D.; Report of the Committee on the Epidemics of Ohio, by George Mendenhall, M. D.; Report of the Committee on Medical Literature, by A. B. Palmer, M.D.; Report of the Special Committee on Medical Education, by James R. Wood, M. D.; Report on Spontaneous Umbilical Hemorrhage of the New-Born, by J. Foster Jenkins, M.D., Yonkers, N. Y.; Report on the Influence of Marriages of Consanguinity upon Offspring, by S. M. Bemiss, M.D., Louisville, Ky.; Report on the Functions of the Cerebellum, by E. Andrews, M.D., Chicago, Ill.; Report of the Treatment best adapted to each variety of Cataract, with drawings illustrating a case, by Mark Stephenson, M.D., Surgeon to the N.Y. Opthalmic Hospital; Report on the Medical Jurisprudence of Insanity, by C. B. Coventry, M.D., Utica, N. Y.; Report on the Law of Registration of Births, Marriages and Deaths, by Edward Jarvis, M. D., Dorchester, Mass.; Report on the Nervous System in Febrile Diseases and the Classification of Fevers by the Nervous System, by Henry Fraser Campbell, M.D., Prof. of Anatomy in Medical College of Georgia; Report on Moral Insanity in its relation to Medical Jurisprudence, by D. Meredith Reese, M. D., L.L.D., etc., of N.Y.; Report on Stomatitis Materna, by D. L. McGugin, A. M., M. D., Keokuk, Iowa; Report on the True Position and Value of Operative Surgery as a Therapeutic Agent, by S. B. Flint, M. D., Louisville, Ky.; a Method for Preserving Membranous Pathological Specimens, by R. D. Arnold, M.D., Savannah, Georgia; Letter from E. D. Fenner, M.D., to Paul F. Eve, M.D., President of the American Medical Association; Prize Essay, "The Clinical Study of the Heart Sounds in Health and Disease," by Austin Flint, M.D., Buffalo, N. Y.—*Clinica Clinice Demonstranda;* Prize Essay, "Vision, and some of its Anomalies, as revealed by the Ophthalmoscope," by Montrose A. Pallen, M.D., of St. Louis, Mo.—*Dux Hominum Medicus Est;* the Plan of Organization, Code of Medical Ethics, and List of Permanent Members, end the volume.

There is a great deal of valuable information contained in this issue, and it should not only be read but be in the possession of every member of our profession who desires to be classed as intelligent. This reminds me of the fact that anybody may buy a copy of the Transactions by forwarding three dollars to Casper Wistar, M.D., Treasurer of the Association, at Philadelphia. Its sale is not confined to the membership, as has been erroneously supposed by many. The Association meets first Tuesday in May next, at Louisville, Ky., and so near, that we hope large numbers of readers will take this opportunity of attending. At some not very distant time we will recur to this treasure of knowledge and make some kind of gleanings for the readers of the *Journal;* meantime we advise every one to take steps to procure a copy for himself.

Wilson's Human Anatomy. A new and improved edition, from an enlarged London edition. Edited by Wm. H. Gobrecht, M.D., Professor of Anatomy in the Philadelphia College of Medicine, etc. With three hundred and ninety-seven illustrations on wood. Philadelphia: Blanchard & Lea. 1858.

All that is necessary in noticing another edition of this favorite work of students of anatomy is to say that it is improved. It has been more generally employed in teaching and studying anatomy than any other book since our recollection of and participation in them.

Dr. Gobrecht has performed his duty well, and will receive the thanks of his readers.

We think that it will be some time before Wilson's Anatomy will be crowded out of dissecting rooms.

Diseases of the Urinary Organs. A complete compendium of their diagnosis, pathology and treatment. By William Wallace Morland, M.D., Fellow of the Massachusetts Medical Society; Member of the Boston Society for Medical Improvement; one of the Attending Surgeons at the Central Office at the Boston Dispensary. With Illustrations. Philadelphia: Blanchard & Lea. 1858.

Dr. Morland informs us that this book is mainly composed of the substance of two prize essays presented to the Boylston Medical Committee. It is as it purports to be, a digest of urinary pathology. The pathology and therapeutics of the urinary organs, commencing with the supra-renal capsules above and ending with the urethra, are fairly represented; and we have no doubt it is as useful a book on the subject as the American practitioner can avail himself of. Its convenient yet ample size enables the author to present an inviting volume to the medical public.

Diabetes and other diseases that do not originate in or immediately affect the urinary organs are very properly left out; as also gonorrhœa, chancre, and specific diseases generally. The illustrations are a valuable addition to the rest of the contents in enabling us to judge of the pathological anatomy and morbid products of these organs.

We are indebted to the author, Professor J. Aitkin Meigs, M.D., for "Hints to Craniographers upon the importance and feasibility of establishing some uniform system, by which the collection and promulgation of Craniological Statistics and the exchange of duplicate Crania may be promoted." It is extracted from the Proceedings of the Academy of Natural Sciences of Philadelphia, of which he is the librarian. The subject of ethnology is becoming a very interesting one, and Dr. Meigs is an able and enthusiastic cultivator of the rich field of research which it affords.

4

"A Summary of the Transactions of the College of Physicians of Philadelphia" is issued in pamphlet form, and contains much very interesting and profitable matter. It contains an abstract of some of the choicest productions and inventions of the able members of that body. Its contributors are such men as Hays A. Hewson, Keating, Stille, Reuschenberger, Corson, Neill, Coates, Henry Bond, etc., who never spend their time in trifles. We may at some future time make extracts or other notice of the contents.

We have received the "Transactions of the Indiana State Medical Society, at its ninth annual session, held in the city of Indianapolis, in May, 1858." They contain several condensed yet valuable papers from the enterprising cultivators of medicine in the State of Indiana. Among them are reports from Dr. Elliott, of Indianapolis, on Chronic Diseases of the Brain; on Microscopy, by Dr. Hutchinson, of Mooresville; and Dr. Calvin West, of Hagerstown, on the same subject; and one on the Uses and Abuse of Mercury, by Dr. N. Knepplar, of Indianapolis. There are also reports of many cases which will be found quite interesting and worthy of careful perusal. We are glad to see that this intelligent assembly of medical men are paying attention to the subject of medical education, and we confidently expect from Drs. W. R. Smith, Newland, Fry and Fishback, the committee, a report next year that will be worthy of so noble and important a subject.

We are also in possession, from the authors of the "Introductory Addresses," one delivered before the medical class of the medical department of the Nashville University, by J. Berrien Lindsley, M.D., Chancellor of the University on Medical Colleges; one by J. W. Benson, M.D., Professor of Anatomy in the medical department of the University of Louisville; and one by J. W. Freer, M.D., Professor of Anatomy in Rush Medical College. They are all respectable productions, and must have been highly entertaining to the classes before which they were delivered.

Professor John H. Rauch, President of the Iowa State Medical Society, has placed upon our table the "Constitution, By-Laws and Code of Ethics of that Institution, with the Transactions of the years 1857 and 1858." It contains the address of Dr. John Liveter, retiring President, which is recommended by the two excellent qualities, brevity and sound sense. There are no other original papers, which we hope will not be said of next year's Transactions.

We have also received an elaborate and practical inaugural dissertation on Strychnia, presented to the faculty of McGill Medical College, Montreal, Canada, prior to receiving the degree of Doctor of Medicine and Surgery, by Alexander P. Reid. This thesis was adjudged worthy of publication by the faculty—which, by the way, we should be glad all thesis were. It certainly manifests a great deal of industry and scientific attainments in its author, and promise for him a useful if not brilliant future.

The Illinois Teacher, organ of the State Association. We have received the December No. of this valuable educational journal. It is quite equal to any of its excellent predecessors. Mr. N. Bateman, its accomplished and untiring editor, has done battle worthy of the all-important cause in which he has been engaged. He takes a manly and independent leave of his numerous readers and editorial confreres. The approaching session of the Teachers' Association on December 28th will have selected his successor before this paragraph meets the eye of our readers. We hope their choice will be marked by the wisdom which prompted them when they selected Mr. Bateman. Meantime we cannot refrain from wishing them and their ex-editor God-speed, and advise all who feel interested in the cause of education to encourage it by subseribing for this able periodical. It is published at Jacksonville, Illinois, at the extremely low rate of one dollar a year in advance. Now is the time to subscribe to begin with the forthcoming volume.

